# *Starmerella bacillaris* Released in Vineyards at Different Concentrations Influences Wine Glycerol Content Depending on the Vinification Protocols

**DOI:** 10.3390/foods12010003

**Published:** 2022-12-20

**Authors:** Chiara Nadai, Vinícius da Silva Duarte, Jacopo Sica, Simone Vincenzi, Milena Carlot, Alessio Giacomini, Viviana Corich

**Affiliations:** 1 Department of Land, Environment, Agriculture and Forestry (TESAF), University of Padova, 35020 Legnaro, Italy; 2 Interdepartmental Centre for Research in Viticulture and Enology (CIRVE), University of Padova, 31015 Conegliano, Italy; 3 Faculty of Chemistry, Biotechnology, and Food Science, The Norwegian University of Live Sciences, 1430 Ås, Norway; 4 Department of Agronomy Food Natural resources Animals and Environment (DAFNAE), University of Padova, 35020 Legnaro, Italy

**Keywords:** non-*Saccharomyces*, alcoholic fermentation, sequential inoculum, wine quality, biocontrol

## Abstract

*Starmerella bacillaris* is a non-*Saccharomyces* yeast proposed for must fermentation together with *Saccharomyces cerevisiae* because of its high glycerol and moderate volatile acidity production. Furthermore, it was demonstrated that the same *S. bacillaris* strains that possess interesting technological properties exhibited antifungal activity against *Botrytis cinerea*, suggesting the release of this yeast in the vineyard. To obtain a positive effect during the following winemaking process, the maintenance of suitable concentrations of *S. bacillaris* is essential. Therefore, to obtain information on the survival of *S. bacillaris*, a small-scale field trial was performed. One week before the harvest, two different concentrations of *S. bacillaris* (10^6^ and 10^7^ cells/mL) were sprayed on Pinot grigio bunches, and the strain concentration was monitored by means of qPCR during the subsequent fermentation process. In addition, the combined effect of different winemaking techniques was evaluated, i.e., the vinification of juice, juice with marc and cryomaceration treatment. Results demonstrated that, under the tested conditions, *S. bacillaris* released in the vineyard remained viable for one week on grape bunches and increased glycerol content during the subsequent fermentation process. Different vinification protocols influenced cell concentrations. In particular, the cryomaceration treatment, due to the use of low temperature, supported *S. bacillaris* growth due to its cryotolerant aptitude. The collected data open new perspectives on the control of alcoholic fermentation, involving both vineyard and cellar management.

## 1. Introduction

*Starmerella bacillaris* (formerly *Candida zemplinina*) is a non-*Saccharomyces* yeast present in the microbial community of grape surfaces and, generally, of oenological environments [1,2,3]. It has also been isolated from fruits, fruit-associated insects and soil [4]. *S. bacillaris* tolerates low temperatures, is able to grow at high sugar concentrations [5], shows strong fructophilic character, high glycerol content, low ethanol content and moderate volatile acidity production [6,7,8,9]. Several studies have highlighted its ability to enhance wine flavour and mouthfeel sensations [7,10,11,12]. Recently, its positive effect on wine protein stability has been proven [13,14]. In recent years, the genomes of several *S. bacillaris* strains have been sequenced [15,16,17,18] and this approach has contributed to the increase in knowledge on important technological properties in winemaking, including glutathione metabolism [19,20].

Due to its interesting technological properties, *S. bacillaris* has been proposed for wine production, and its potential use as a co-starter, in both sequential and mixed fermentations with *Saccharomyces cerevisiae*, has been tested [8,21,22,23,24]. When inoculated, at first *S. bacillaris* strains alleviated the osmotic stress of *S. cerevisiae* cells due to the selective fructose consumption [25]. Another benefit to the use of mixed non-*Saccharomyces*–*S. cerevisiae* inoculation is to the reduction of the total sulphur dioxide addition in wine, due to the fact that, generally, non-*Saccharomyces* yeasts are sensitive to SO_2_. In particular, it was shown that 50 mg of total SO_2_ was sufficient to inhibit the growth of *S. bacillaris* [26]. Sulphur dioxide is a chemical preservative used in winemaking that possesses antiseptic, antioxidant and antioxidasic properties [27]. However, sulphites are toxic to organisms, and for this reason consumers ask for the reduction in the use of sulphur dioxide in wines. Therefore, the use of *S. bacillaris* could meet consumer demands for wines with reduced SO_2_ content.

Moreover, it has been demonstrated that the same *S. bacillaris* strains that possessed interesting technological properties exhibited antifungal activity against *Botrytis cinerea* and *Penicillium expansum* [9,21]. The simultaneous presence of antifungal activities and wine technological properties suggests the potential use of *S. bacillaris* as a biocontrol agent, by being released in vineyards, and as a fermenting agent after harvest. Indeed, yeast cells present on the grape berry surface can act as starter to initiate the fermentation process. A preliminary work, focused on *S. bacillaris*’ ability to persist on the grape surface under laboratory scale conditions, demonstrated that the strain sprayed on some grape bunches remained at high concentrations for at least 6 days after the inoculum [28]. Therefore, *S. bacillaris* demonstrated its potential use as a biocontrol agent at harvest time, when no synthetic fungicide treatments are allowed. At the reference concentration responsible for antifungal activity, *S. bacillaris* produced high glycerol concentrations, influencing the final wine quality. Moreover, authors reported positive effects on fermentation even when the inoculum concentration was 10 times lower than the reference concentration [28]. 

These preliminary results encouraged open field trials to evaluate *S. bacillaris* activity in vineyards and in the grape juice during the post-harvest vinification process. Information on the survival of *S. bacillaris* on the surface of the grape berries is crucial to maintain a suitable concentration of *S. bacillaris* in the vineyard; essential to obtaining a positive effect during the winemaking process.

At the moment these data are missing, as well as those related to the effects of different winemaking practices.

In this study, a small-scale field trial was performed to evaluate *S. bacillaris* survival after vineyard inoculation. One week before harvest, two different concentrations of *S. bacillaris*, 10^6^ and 10^7^ cells/mL, were sprayed on Pinot grigio bunches to evaluate the effect of direct bunch inoculation on the subsequent fermentation process in terms of glycerol production. In addition, the combined effects of different winemaking techniques were also evaluated: vinification of juice, juice with marc or cryomaceration. Fermentation trials were conducted, taking advantage of the presence of *S. bacillaris* on the grapes, inoculating *S. cerevisiae* after 48 h. Grape juice and must originating from untreated grape samples were immediately inoculated with *S. cerevisiae* and used as controls.

## 2. Materials and Methods

### 2.1. Yeast Strains and Growth Conditions

The yeast strains tested in this work were *S. bacillaris* FRI751 [16], collected from dried grapes of the Raboso Piave variety as previously described [21], and the commercial wine strain *S. cerevisiae* EC1118 (Lallemand Inc., Montreal, Canada). 

Pre-culture of the *S. bacillaris* strain used in this work was prepared as described by Bovo et al. [29]. The concentration of *S. bacillaris* in a stationary phase YPD (yeast extract–peptone–dextrose, Difco, Milan, Italy) culture was determined by flow cytometry count using a CyFlow SL flow cytometer (Partec, Münster, Germany), following the manufacturer’s instructions. The culture medium was removed by centrifugation and the cell pellet was re-suspended in a volume of NaCl (0.9% *w*/*v*) physiological solution, in order to achieve 3 × 10^8^ cells/mL.

### 2.2. Yeast Release in the Vineyard and Experimental Procedure

**Figure 1:**
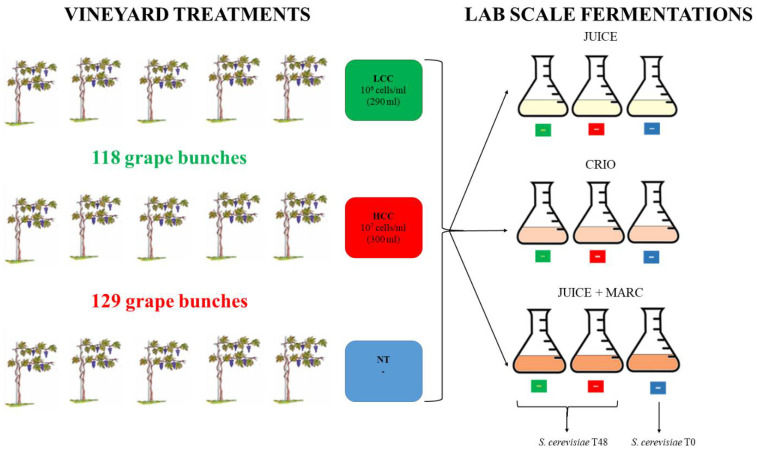
Experimental procedure.

In a vineyard, 15 plants of Pinot grigio variety were selected to be used for the release of *S. bacillaris* at different concentrations. The plants were split in three groups: five plants were used as the untreated control (NT), five plants were treated with a physiological solution containing 1 × 10^6^ cells/mL of *S. bacillaris* (low cell concentration, LCC) obtained diluting the yeast pre-culture (3 × 10^8^ cells/mL concentration) and five plants were treated with a physiological solution containing 1 × 10^7^ cells/mL of *S. bacillaris* (high cell concentration, HCC), obtained by the same pre-culture (Figure 1).

One week before harvest, the two yeast solutions were sprayed on the grape bunch only with a hand pump, making sure to cover the whole surface. At the end of the treatment, by measuring the remaining volume of each cell suspension, 300 mL was sprayed on 129 grape bunches from the five plants of the HCC trial (10^7^ cells/mL suspension), while 290 mL was sprayed on 118 grape bunches from the five plants of the LCC trial (10^6^ cells/mL suspension). The remaining five plants, used as controls (NT), were not sprayed.

One week after the yeast release in the vineyard, the harvest was performed. For each treatment, 400 g of bunches were collected in triplicate and washed with peptone water to perform microbiological analyses. 

The remaining bunches were used for vinification. For each treatment (NT, HCC and LCC), three vinification protocols were performed: grape juice fermentation (JUICE) that mimics white wine fermentation protocols, cryomaceration O.N. of juice and skins at 5 °C followed by juice fermentation after skin removal (CRYO) and fermentation of juice with skins (JUICE + MARC) that mimics red wine fermentation protocols.

### 2.3. Fermentation Trials

Pre-cultures of the *S. cerevisiae* strain used in this work were prepared as described by Bovo et al. [29]. Fermentations were performed in 250 mL-capacity Erlenmeyer flasks sealed with a silicon cap and supplied with a bowed glass pipette containing 200 mL of grape juice. 

In LCC and HCC, the inoculum of *S. cerevisiae* (approximately 1 × 10^6^ cells/mL) was performed forty-eight hours after grape crushing. A single strain fermentation with *S. cerevisiae* EC1118 was used as a control where juices were immediately inoculated with *S. cerevisiae*.

All the flasks were kept at 18 °C until the end of fermentation. All experiments were performed in triplicate. Alcoholic fermentation was monitored by measuring the weight loss twice a day during the whole fermentation process. Each fermentation was stopped when the weight loss was lower than 0.1 g after 24 h. 

At the beginning of fermentation, after 48 h and at the end of fermentation, an aliquot of all samples was collected, centrifuged and the pellet was washed with water and frozen for molecular analyses, while the supernatant was used for the chemical analyses.

### 2.4. Microbiological Analysis

Total yeast quantification was performed by plate count in YM Agar medium (yeast extract–malt extract–peptone–dextrose–agar, Difco, Milan, Italy) and bacteria quantification was performed by plate count in PCA medium (plate count agar, Difco, Milan, Italy). Chloramphenicol (10 mg/mL) was added to YMA to prevent the growth of bacteria, while to prevent the growth of yeasts and moulds, nystatin (5 mg/mL) was added to PCA. Ten-fold dilutions of samples in Ringer’s solution (Oxoid, Milan, Italy) were spread onto plates. Plates were incubated at 28 °C for 3 days before plate count.

### 2.5. Chemical Analysis

Ammonia and amino nitrogen were measured enzymatically using commercially available enzymatic kits from Steroglass (Steroglass, San Martino in Campo, Italy) according to the manufacturer’s instructions in the Hyperlab multi-parametric analyser. 

HPLC was used to determine the concentrations of glucose, fructose, acetic acid, glycerol and ethanol, as described by Lemos Junior et al. [8]. Ten microlitres of filtered sample was analysed using a Waters 1525 HPLC binary pump (Waters, Milford, MA, USA) equipped with a 300 mm × 7.8 mm stainless steel column packed with an Aminex HPX-87H HPLC column (Bio-Rad, Hercules, CA, USA). A Waters 2414 refractive index detector (Waters, Milford, MA, USA) set at 600 nm wavelength was used for the determination of glucose, fructose, glycerol and ethanol, while the determination of acetic acid and succinic acid was obtained by using a Waters 2487 dual absorbance detector (Waters, Milford, MA, USA) set to 210 nm. The analyses were performed isocratically at 0.6 mL/min and 65 °C with a cation exchange column (Aminex HPX-87H) and a Micro-Guard Cation H+ Cartridge (Bio-Rad Laboratories, Hercules, CA, USA), using 0.01 N H_2_SO_4_ as the mobile phase. Calibration curves of the individual compounds were used to calculate their concentrations, expressed as g/L or % *v*/*v* in the samples, through the determination of the peak area by the Breeze (Waters, Milford, MA, USA) programme. 

### 2.6. Real Time PCR Quantification of Starmerella Bacillaris 

Total DNA was extracted using the DNeasy PowerSoil Pro Kit (Qiagen, Hilden, Germany) according to the manufacturer’s instructions. Quality and quantity of the extracted DNA were determined with Tecan’s NanoQuant Plate (Tecan group, Männedorf, Switzerland). DNA concentration was determined by measuring the absorbance at 260 nm, while DNA quality was estimated from the A260/A280 ratios.

Real-time PCR was carried out on a CFX96 cycler real-time PCR detection system (Bio-Rad Laboratories, Inc., Hercules, CA, USA) in white-walled PCR plates (96 wells) using SsoFast EvaGreen Master Mix (Bio-Rad Laboratories, Inc., Hercules, CA, USA) as described by Nadai et al. [30], with some modifications. The combined primer annealing/elongation step was set at 62 °C for 10 s.

To detect the presence of the *S. bacillaris* strain FRI751, specific primers were designed (Table 1) which, based on the sequence of its genome, were amplified for a unique region of this strain. It was verified that these primers do not amplify either with other *Starmerella* strains or with other oenological yeasts (Figure S1).

A calibration curve was constructed using *S. bacillaris* FRI751 DNA. Cell samples that were used for DNA extraction were prepared as follows: tubes containing 10^6^ cells of *Hanseniaspora uvarum* type strain CBS104 (Centraalbureau voor Schimmelcultures, Utrecht, Netherlands) were re-suspended in Pinot grigio grape juice and ten-fold dilutions of a cell culture of *S. bacillaris* were added in order to obtain a final concentration from 10^2^ to 10^6^ cells. Tubes were centrifuged, DNA was extracted as previously described and used to construct the calibration curve. In this way, the presence of contaminants, such as tannins and other polyphenols, on the efficiency of the qPCR reaction was taken into account [31]. Moreover, it was possible to verify the presence of any variations in the extraction efficiency due to the different concentrations of the cells, evaluating the linearity of the observations. The equation of the curve (R^2^ = 0.995) was used to estimate the amount of *S. bacillaris* present in the various sampling points. 

### 2.7. Statistical Analysis

XLSTAT software vers. 2016.02, Addinsoft (Paris, France) was used to perform the statistical data analysis. Data were subjected to the analysis of variance (one-way ANOVA) followed by the Tukey’s post hoc test. The averages of three independent replicates were considered, and *p*-values lower than 0.05 were chosen to identify statistically significant differences among the samples. 

## 3. Results and Discussion

### 3.1. Release of Starmerella Bacillaris in Vineyard and Inoculum Evaluation

A small-scale field trial was performed releasing *S. bacillaris* into a vineyard one week before harvest at two different concentrations: high cell concentration, HCC (10^7^ cells/mL), and low cell concentration, LCC (10^6^ cells/mL). The HCC was selected as it is the standard cell concentration suggested by bioactive yeast manufacturers and used by other authors in biocontrol experiments [32,33,34]. The LCC was tested to verify if a lower concentration of yeast was enough to allow *S. bacillaris* to persist on grapes. 

The ability of the yeast to colonise the bunches is of fundamental importance to obtain a suitable *Starmerella* concentration that contributes to the fermentation process until the moment of harvest.

One week after the treatment, the bunches were harvested to carry out fermentation trials and to verify the influence of different *S. bacillaris* concentrations sprayed in the vineyard on the vinification. This length of time, from the inoculation of *Starmerella* to the crushing of the bunches, was chosen based on previous laboratory tests which have shown that the amount of *S. bacillaris* sprayed on the bunch remained constant up to one week after the treatment [28].

Different vinification protocols (JUICE, CRYO and JUICE + MARC) were set up to investigate the influence of the main oenological treatments on the grapes on the detachment of *S. bacillaris* cells from the surface of the berries, and therefore on the increase in glycerol concentration in wine. These treatments included the standard vinification practice used for Pinot grigio wine production: the grapes are crushed and immediately pressed for juice fermentation (JUICE). 

In each vinification protocol, the untreated control (NT) was immediately inoculated with *S. cerevisiae*. This allowed the quantification of the microbial population naturally present on the grapes (T0) and to compare a standard fermentation procedure (*S. cerevisiae* inoculated immediately after crushing/maceration) with the new one that inoculates with *S. cerevisiae* 48 h after crushing/maceration of *S. bacillaris*-treated grapes. 

**Figure 2:**
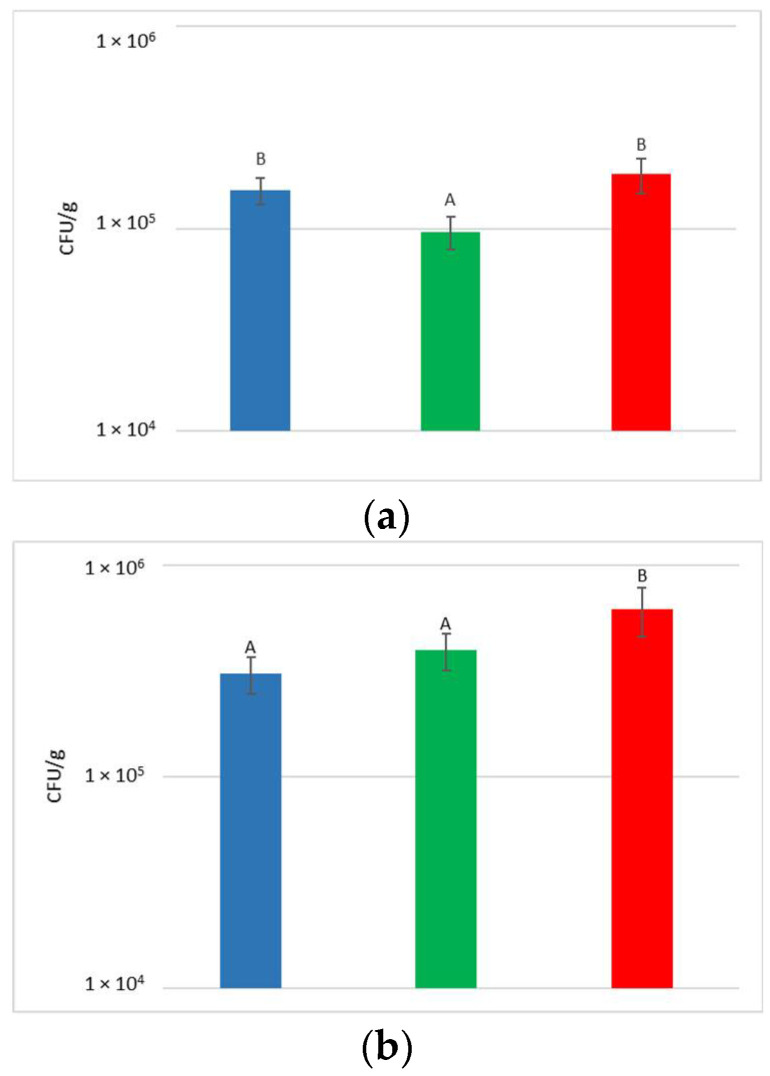
Total yeast (**a**) and bacteria (**b**) concentrations present on grape surfaces one week after the release of *S. bacillaris* in the vineyard. NT: untreated control (blue), LCC: low cell concentration (green), HCC: high cell concentration (red). Data are expressed as the average of three replicates ± standard deviation. For each figure, different letters indicate significant differences between values (*p* = 0.05).

At the harvest time, the concentrations of microbial populations (bacteria and yeasts) present on grapes surface were determined after washing the bunches to recover the microorganisms present on the surface (Figure 2).

With regard to the yeast, their concentration on the bunches varied from 9.7 × 10^4^ to 1.9 × 10^5^ CFU/g, with an average of 1.5 × 10^5^ CFU/g. Despite the limited variability recorded between the treatments, a significantly higher yeast concentration was measured in NT and HCC with respect to LCC. 

At the time of the vineyard treatment, each grape bunch, weighing an average of 90 g, was treated with 2.36 ± 0.08 mL of *S. bacillaris* solution. Taking into account that part of the solution will not adhere to the grape bunch, repeated spraying tests allowed to quantify 0.6 mL as the volume that remains on the bunch surface. Therefore, an estimate of the adherent cells per gram on the grape bunch was about 6 × 10^3^ in the LCC sample and 6 × 10^4^ in the HCC sample. These results are in accordance with recent work that evaluated two bioactive yeasts (*Metschnikowia pulcherrima* and *Aureobasidium pullulans*) in field experiments [32]. Agarbati and colleagues used a solution containing 10^7^ cells/mL of yeast in a field during pre-harvest treatments and, both before and after the treatment, they found a yeast concentration of about 2–8 × 10^4^ CFU/mL on the grapes.

The concentration of total population measured in NT was notably higher than that of *Starmerella* released with the treatment in the vineyard, even in HCC. For this reason, no increase in the total yeast population was observed, and the different concentrations measured were due to random variations in the populations present on the single bunches. 

The concentration of bacteria present on the surface of the bunches varied from 3.1 × 10^5^ to 6.2 × 10^5^ CFU/g. Statistical analysis shows that HCC had a significantly higher concentration of bacteria than LCC and NT. 

### 3.2. Fermentation Trials

To evaluate the fermentation performance, fermentation trials were set up on a laboratory scale. One week after the *Starmerella* release in the vineyard, the bunches from each treatment were separately harvested, crushed and de-stemmed. 

The initial composition of all musts and grape juices, measured in total sugars, ammonia and amino nitrogen, is reported in Table 2. 

The analysis of the total sugars highlighted significant differences between the samples, as sugars varied from 188.3 to 198.7 g/L. Regarding the yeast assimilable nitrogen concentrations (92.7 mg/L–127.2 mg/L), the amino nitrogen concentrations varied from 64.4 to 78 mg/L and those of ammonia nitrogen varied from 28.3 to 49.3 mg/L. The limited differences registered (generally about 5%) depended mainly on the maturity level of the collected bunches. No effect of the treatments was observed on juice must composition, as no significant differences are attributable neither to the *S. bacillaris* treatment nor to the vinification protocol. 

In NT, the *S. cerevisiae* EC1118 strain was immediately inoculated, while in HCC and LCC the inoculum was performed 48 h after the start of fermentation in order to favour *S. bacillaris* development.

The fermentation kinetics of HCC and LCC were compared with those of NT, to determine how the simultaneous presence of the two species could influence the fermentation process.

**Figure 3:**
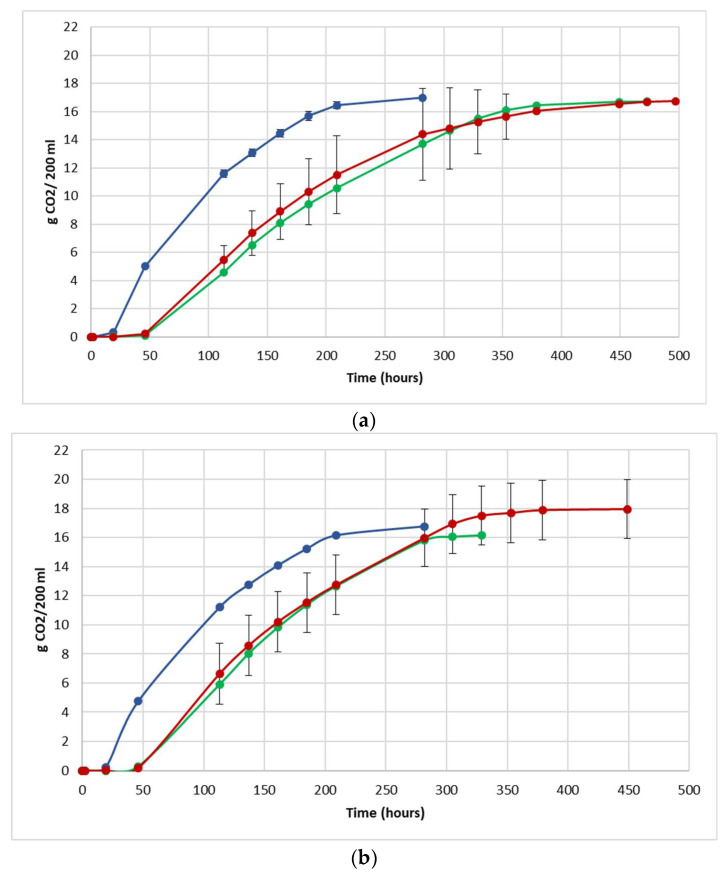

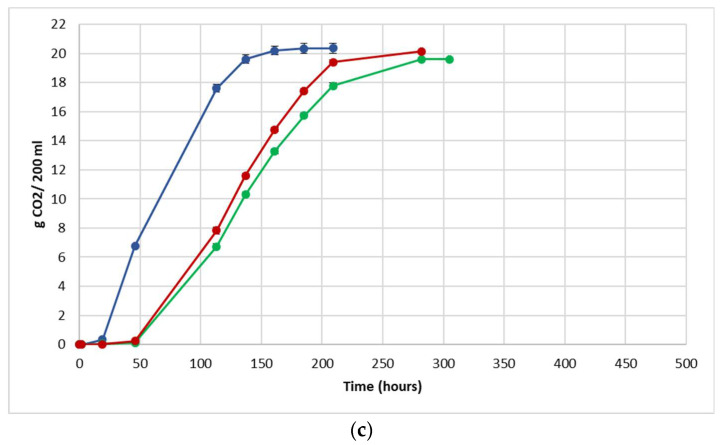
Fermentation kinetics (CO_2_ released/time) in natural grape must. (**a**) JUICE, (**b**) CRYO and (**c**) JUICE + MARC. NT: untreated control (blue), LCC: low cell concentration (green), HCC: high cell concentration (red). Data are expressed as the average of three replicates ± standard deviations.

The fermentation performance was followed by daily monitoring of the decrease in weight of the flasks, due to the loss of CO_2_ produced during fermentation (Figure 3). 

For each vinification protocol, as expected, NT showed the fastest kinetics, completing sugar consumption earlier than LCC and HCC. The fermentation kinetics of HCC and LCC were very similar, regardless of the fermentation protocol. The fermentations ended after between 12 and 19 days in JUICE and CRYO and between 9 and 13 days in JUICE + MARC, confirming that the presence of the skins speed up the fermentation process. 

In HCC and LCC, before *S. cerevisiae* inoculation, a very low CO_2_ production was registered and after inoculation, the overall fermentation rates appeared to slow down due to the presence of the vineyard population. This fermentation trend is generally observed when *S. bacillaris* is used in sequential inoculation with *S. cerevisiae* in both natural and synthetic must fermentations [21,28,35]. A previous study suggested that in sequential fermentations with *S. bacillaris*, the reduction in growth rate of *S. cerevisiae* is probably due to the high nutrient consumption before the inoculation of *S. cerevisiae* [35,36]. 

After 48 h from the start of fermentation, before the addition of *S. cerevisiae* in LCC and HCC fermentations, total sugars and ammonia and amino nitrogen concentrations were measured. Residual sugars varied between 112 g/L and 187.3 g/L. The residual amino nitrogen varied between 0 mg/L and 67.4 mg/L, while the ammonia nitrogen residues varied between 3 mg/L and 43.7 mg/L. As expected, NT, where *S. cerevisiae* was inoculated at T0, consumed significantly more sugars than LCC and HCC, while almost all the available nitrogen was depleted.

Considering the level of variability in the initial sugar and nitrogen content, the percentages of consumption, instead of the residual concentrations, are reported in Table 3. 

As expected, regardless the vinification protocol, NT, where *S. cerevisiae* was inoculated at T0, consumed significantly more sugars than LCC and HCC (in particular in JUICE + MARC, the fermentation showing the fastest kinetics), while almost all the available nitrogen was depleted. In general, JUICE + MARC showed a lower nitrogen consumption compared to the other vinification protocols, this could be due to a gradual nitrogen release from the part of the pulp generally stuck to the skins that occurred during the fermentation.

Despite the different fermentation times, the transformation of sugar into ethanol was completed in all the vinifications (sugar residue <1 g/L). At the end of fermentation, glycerol, acetic acid and ethanol concentrations were measured (Table 4). The end of fermentation is the time when the last CO_2_ measurement was collected, shown in Figure 3.

The final ethanol concentrations varied between 11.8 and 12.7% *v*/*v*, simply reflecting the different initial sugar content. In some studies, *S. bacillaris* used in sequential fermentations has demonstrated the ability to reduce ethanol content by up to 1% in presence of high concentrations of yeast (10^7^ cells/mL) and sugars (240 g/L) [37]. In the present study, the effect of the treatments on ethanol content was not appreciable. This could be due to both the limited sugar content and a lower concentration of *S. bacillaris* released in the vineyard. 

Acetic acid production was limited, and values were quite variable (from 0.09 to 0.50 g/L). In JUICE and JUICE + MARC, NT acetic acid concentration showed no significant differences from LCC and HCC, respectively. However, the concentrations are acceptable and lower than the acetic acid detectable level, 0.7–0.9 g/L [38]. 

Glycerol concentrations varied between 5.8 and 9.1 g/L. The glycerol content in HCC was always significantly higher than NT. Considering LCC in JUICE and JUICE + MARC, no significant differences were found with respect to NT, while CRYO resulted in a significantly higher production of glycerol compared to NT. This finding evidenced that wines obtained from bunches treated with *S. bacillaris* in high concentration resulted in higher glycerol content than the untreated control, regardless of the vinification protocol. High glycerol content always occurs when *S. bacillaris* is inoculated in cellar during sequential fermentations with *S. cerevisiae* in both natural and synthetic must [14,21,22,24,28,37]. 

Glycerol concentration is proportional to the sugar content, as it is produced as a response to hyperosmolarity [39]. Due to the variability in the initial sugar content, the ratio between the glycerol produced and the sugars consumed (glycerol/sugar ratio) was calculated and compared for each vinification protocol (JUICE, CRYO and JUICE + MARC) and the results are reported in Table 5.

Taking into account the initial sugar content, NT always produced a low ratio, regardless of the vinification protocol. Considering the inoculum size of *S. bacillaris* in the vineyard, JUICE HCC showed a significantly higher ratio than LCC and NT. In CRYO and JUICE + MARC, LCC and HCC showed a similar ratio, significantly higher than NT. Due to different initial sugar levels, in JUICE + MARC LCC, the ratio showed significantly higher glycerol production than NT, despite the fact that the glycerol content in the wine was not significantly different from NT. Generally, a significantly higher quantity of glycerol than NT was found even in the trials where *S. bacillaris* was released at low concentration. This finding is in accordance with previous work, where a positive effect on glycerol production was achieved during sequential fermentation at inoculum concentrations of 10^4^ cells/mL; 100 times less than the conventional *S. bacillaris* inoculum [28]. 

### 3.3. Quantification of Total Yeasts and Starmerella Bacillaris

**Figure 4:**
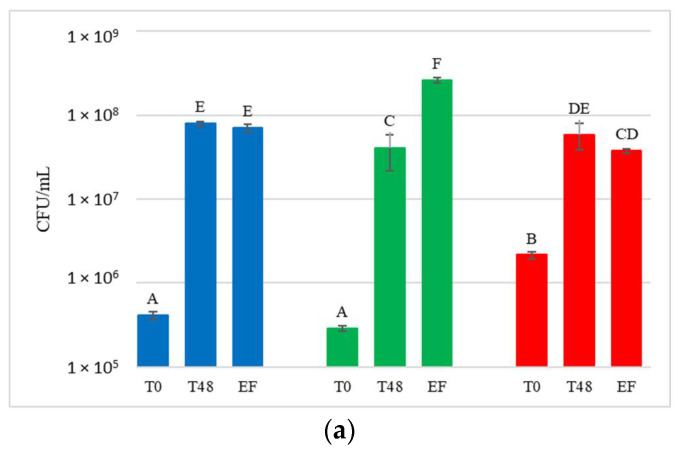

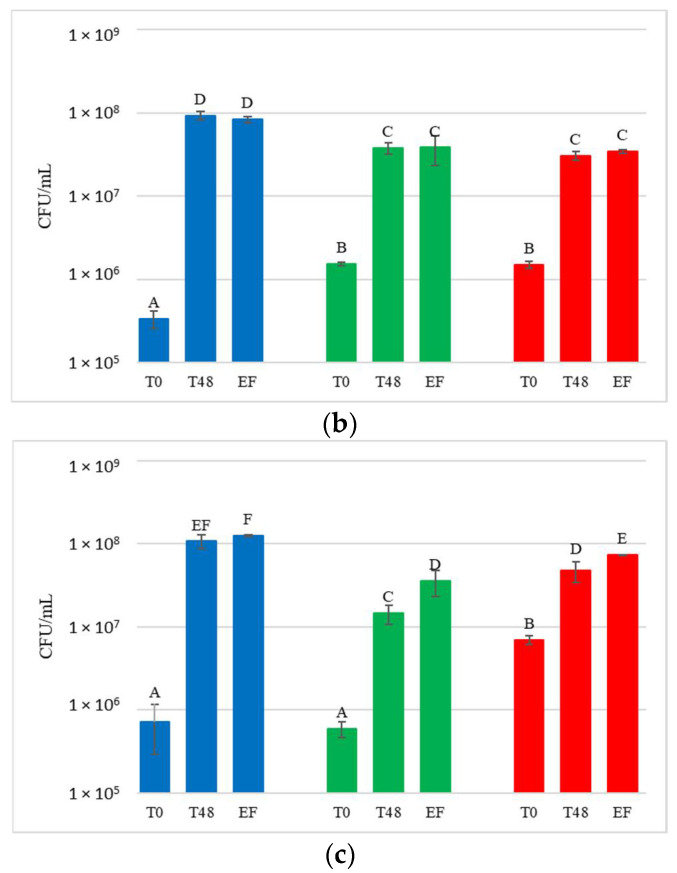
Total yeast concentrations measured during the fermentation in natural grape must. (**a**) JUICE, (**b**) CRYO and (**c**) JUICE+MARC. NT: untreated control (blue), LCC: low cell concentration (green), HCC: high cell concentration (red). Data are expressed as the average of three replicates ± standard deviations. For each figure, different letters indicate significant differences between values (*p* = 0.05).

For each treatment (NT, HCC and LCC), the concentration of total yeast in the must or juice (T0) after 48 h (T48) and at the end of fermentation (EF) was determined by plate count (Figure 4).

Yeast present in the initial musts and juices (T0) showed very variable concentrations, ranging from 2.9 × 10^5^ to 7 × 10^6^ CFU/mL. Comparing these results with the concentration of yeast present on the grape surface at harvest (9.7 × 10^4^ to 1.9 × 10^5^ CFU/g of bunch), a general increase in the total population immediately after crushing was observed. At T0, NT showed an average concentration of 4.9 × 10^5^ CFU/mL and HCC always had a significantly higher yeast concentration than NT (on average 3.6 × 10^6^ CFU/mL). LCC, on the other hand, showed a significantly higher yeast concentration than NT only in CRYO.

After 48 h, before the inoculation of *S. cerevisiae* in HCC and LCC, the total number of yeast present in the musts varied between 1.5 × 10^7^ CFU/mL and 1.1 × 10^8^ CFU/mL, indicating that, compared to T0, a significant increase in total yeast population was always observed.

At the end of fermentation, yeast concentration varied between 3.4 × 10^7^ CFU/mL and 2.6 × 10^8^ CFU/mL, and was not significantly different from that at 48 h.

**Figure 5:**
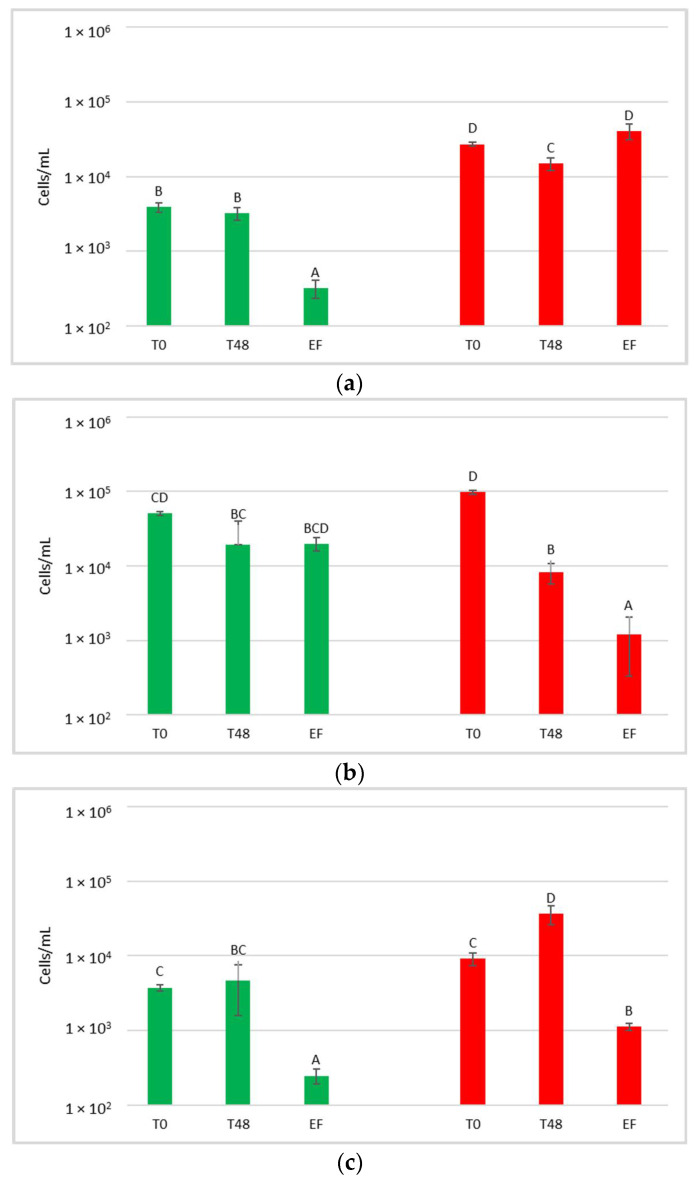
*Starmerella bacillaris* concentrations measured during fermentation of natural grape must. (**a**) JUICE, (**b**) CRYO and (**c**) JUICE + MARC. LCC: low cell concentration (green), HCC: high cell concentration (red). Data are expressed as the average of three replicates ± standard deviations. For each figure, different letters indicate significant differences between values (*p* = 0.05).

The dynamics of the *S. bacillaris* population released on the grapes in the vineyard was investigated by real-time PCR during fermentation. A calibration curve, constructed with the specific primers designed for *S. bacillaris* FRI751, allowed the absolute quantification of *S. bacillaris* in each sample. This quantification is fundamental to verify whether *S. bacillaris* cell concentration supported the glycerol/sugar ratio values found in the different trials. Figure 5 shows the trends over time in the *S. bacillaris* concentrations during fermentation.

**Figure 6:**
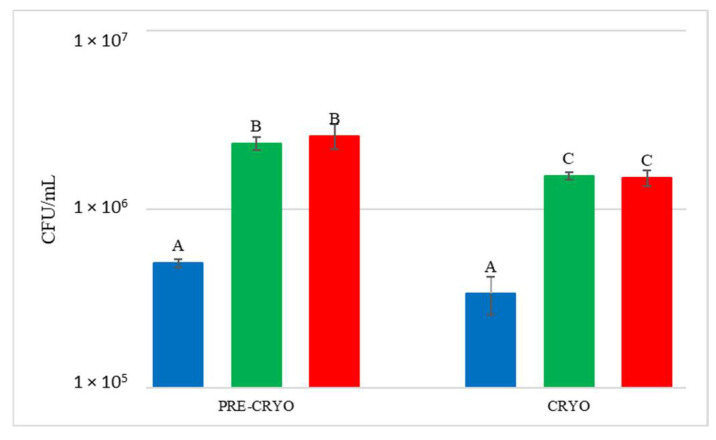
Total yeast count before (PRE-CRYO) and after (CRYO) cryomaceration. NT: untreated control (blue), LCC: low cell concentration (green), HCC: high cell concentration (red). Data are expressed as the average of three replicates ± standard deviations. Different letters indicate significant differences between values (*p* = 0.05).

DNA from NT samples were amplified using *S. bacillaris* primers as well, and results showed the presence of *S. bacillaris* in concentrations from 10^1^ to 10^2^ cells/mL. This finding could be due to the limited contamination of vine plants used as control during the cell spraying treatment. The fermentation of the treated grape bunches evidenced, as expected, the presence of *S. bacillaris* at higher levels in HCC than in LCC, except for CRYO, where the concentrations were comparable. To verify the effect of cryomaceration on yeast population, plate counts were carried out both before (PRE-CRYO) and after (CRYO) cryomaceration (Figure 6).

After the treatment, the total yeast concentration did not increase but remained unchanged or decreased, suggesting that generally this practice does not favour either the release of yeast from the skins or yeast growth. In the other treatments, *S. bacillaris* quantification at T0 showed values similar to the estimated inoculum size.

**Figure 7:**
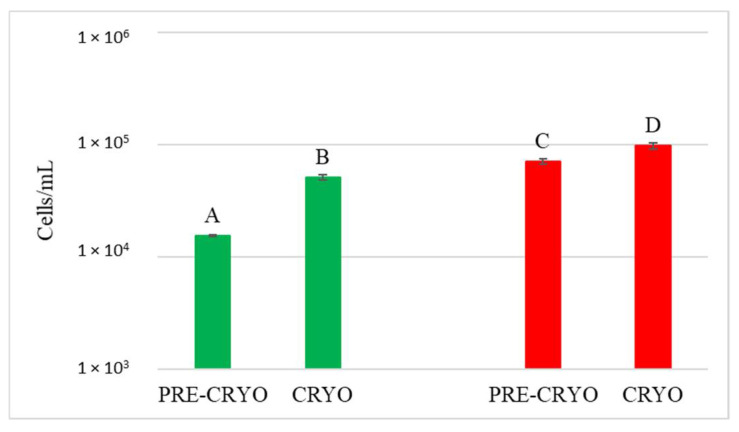
*Starmerella bacillaris* concentrations measured before (PRE-CRYO) and after (CRYO) cryomaceration. LCC: low cell concentration (green), HCC: high cell concentration (red). Data are expressed as the average of three replicates ± standard deviations. Different letters indicate significant differences between values (*p* = 0.05).

The quantification of the *S. bacillaris* population was investigated by qPCR (Figure 7).

Surprisingly, in both LCC and HCC samples, *S. bacillaris* concentration significantly increased after cryomaceration, suggesting that cryomaceration favoured *S. bacillaris* growth due to its cryotolerant aptitude.

In JUICE HCC, *S. bacillaris* concentration remained constant at about 10^4^ cells/mL. JUICE LCC showed a lower initial concentration (3.9 × 10^3^ cells/mL) compared to HCC and a significant decrease at the end of fermentation (from 3.2 × 10^3^ cells/mL at 48 h to 3.2 × 10^2^ cells/mL at end of fermentation).

In CRYO LCC the cell concentration did not vary significantly over time, recording an average value of 3 × 10^4^ cells/mL, while CRYO HCC showed high initial cell concentration (9.7 × 10^4^ cells/mL) which decreased over time, reaching 1.2 × 10^3^ cells/mL at the end of fermentation, indicating no *S. bacillaris* growth.

Finally, in JUICE + MARC LCC, an increase in cell concentration was registered in the first 48 h (from 9.2 × 10^3^ to 3.6 × 10^4^ cells/mL) followed by a sharp decrease at the end of fermentation (1.1 × 10^3^ cells/mL), while in JUICE + MARC HCC, where the initial concentration was 3.8 × 10^3^ cells/mL, there was no increase in the first 48 h and a sharp decline at the end of fermentation (from 4.6 × 10^3^ cells/mL at 48 h to 2.5 × 10^2^ cells/mL at the end of fermentation).

Only JUICE LCC showed no significant difference with NT in terms of the glycerol/sugar ratio, although the *S. bacillaris* cell concentration trend was very similar to JUICE + MARC LCC. This result could be due to the influence of the yeast natural population on the *S. bacillaris* strain in terms of glycerol metabolism and production.

## 4. Conclusions

Results reported in this work demonstrated that *S. bacillaris* released in the vineyard remained viable for one week on grape bunches and increased glycerol content during the subsequent fermentation process. 

This small-scale field trial allowed an accurate quantification of the cell fraction of the inoculum that effectively adhered to the grape surface. This information is crucial for the scale-up of future field trials. The strain specific qPCR quantification method demonstrated the presence of *S. bacillaris* during the fermentation process. The differences in terms of cell concentrations were in accordance with the inoculum size, although generally, no *S. bacillaris* growth was reported. Therefore, the concentration of *S. bacillaris* sprayed in the vineyard was crucial in achieving a positive effect on the wine glycerol content. A cellular concentration (LCC, 10^6^ cells/mL) lower than the conventional (HCC, 10^7^ cells/mL) was demonstrated to be effective depending on the vinification protocols. In fact, only the cryomaceration treatment, due to the use of low temperature, supported *S. bacillaris* growth. Despite the glycerol increase, no ethanol reduction was observed, due to the low sugar content in the grapes and the limited concentration of *S. bacillaris* released in the vineyard. Acetic acid production was limited, and always lower than the acetic acid detectable level.

The collected data provide information for modulating *S. bacillaris* concentrations at the start of fermentation and thus the glycerol content in wines. This goal can be achieved in vineyards by choosing a suitable inoculum size and/or repeating the treatment during grape ripening and in the pre-harvest interval. In the cellar, the yeast concentration can be increased using an appropriate vinification protocol.

## Figures and Tables

**Table 1:** Details of primers and amplicons used in this work.

Primer	Sequence (5′-3′)	Tm	Amplicon Length
Sb Fw	ATA CCG ACT GCC ATC TAT C	65 °C	170 bp
Sb Rev	TAA CTG CTA CTG CTA CCT AC	66 °C	

**Table 2:** Initial composition of musts and grape juices.

		Sugars (g/L)	Amino Nitrogen (mg/L)	Ammonia Nitrogen (mg/L)
JUICE	NT	188.33 ± 0.58 A	71.63 ± 1.55 BC	35.33 ± 0.58 B
	LCC	193.67 ± 1.15 B	67.87 ± 1.63 AB	38.67 ± 0.58 D
	HCC	195.67 ± 1.53 BC	74.90 ± 0.44 CD	48.67 ± 0.58 E
CRYO	NT	195.33 ± 1.15 BC	77.97 ± 3.06 D	29.33 ± 0.58 A
	LCC	188.67 ± 1.15 A	74.83 ± 0.86 CD	36.67 ± 0.58 BC
	HCC	188.33 ± 2.08 A	64.37 ± 1.59 A	28.33 ± 0.58 A
JUICE +MARC	NT	195.33 ± 1.15 BC	77.63 ± 1.72 D	37.67 ± 0.58 CD
	LCC	188.33 ± 1.15 A	67.17 ± 1.59 AB	39.00 ± 1.00 D
	HCC	198.67 ± 0.58 C	77.83 ± 2.89 D	49.33 ± 0.58 E

Data are expressed as the average of the replicates ± standard deviations. Different letters in each column indicate significant differences between values (*p* = 0.05). NT: untreated control, LCC: low cell concentration, HCC: high cell concentration.

**Table 3:** Percentages of consumption of sugars and YAN (yeast assimilable nitrogen) after 48 h.

		Sugars	YAN
JUICE	NT	31.67 ± 3.32 B	97.20 ± 0.90 D
	LCC	4.98 ± 2.80 C	38.97 ± 5.14 C
	HCC	4.26 ± 0.75 C	35.07 ± 1.08 BC
CRYO	NT	29.70 ± 1.06 B	97.22 ± 0.86 D
	LCC	4.41 ± 1.19 C	30.50 ± 3.21 BC
	HCC	2.12 ± 1.40 C	26.77 ± 3.45 B
JUICE +MARC	NT	42.66 ± 0.77 A	95.38 ± 1.28 D
	LCC	0.00 ± 0.00 -	0.00 ± 0.00 -
	HCC	6.21 ± 2.47 C	12.64 ± 2.44 A

Data are expressed as the average of the replicates ± standard deviations. Different letters in each column indicate significant differences between values (*p* = 0.05). NT: untreated control, LCC: low cell concentration, HCC: high cell concentration.

**Table 4:** Glycerol, acetic acid and ethanol concentrations at the end of fermentation.

		Ethanol (% *v*/*v*)	Acetic Acid (g/L)	Glycerol (g/L)
JUICE	NT	12.65 ± 0.10 E	0.16 ± 0.01 BC	5.94 ± 0.30 AB
	LCC	12.45 ± 0.02 DE	0.21 ± 0.03 CD	6.31 ± 0.11 AB
	HCC	12.64 ± 0.19 E	0.50 ± 0.02 F	9.11 ± 0.97 E
CRYO	NT	12.55 ± 0.02 DE	0.13 ± 0.01 AB	5.78 ± 0.06 A
	LCC	12.06 ± 0.03 AB	0.47 ± 0.03 EF	7.71 ± 0.19 CD
	HCC	12.31 ± 0.02 BCD	0.43 ± 0.02 E	8.02 ± 0.06 CDE
JUICE +MARC	NT	12.14 ± 0.05 BC	0.24 ± 0.02 D	5.82 ± 0.24 AB
	LCC	11.83 ± 0.16 A	0.09 ± 0.02 A	6.90 ± 0.43 BC
	HCC	12.41 ± 0.09 CDE	0.19 ± 0.02 CD	8.10 ± 0.23 DE

Data are expressed as the average of the replicates ± standard deviations. Different letters in each column indicate significant differences between values (*p* = 0.05). NT: untreated control, LCC: low cell concentration, HCC: high cell concentration.

**Table 5:** Glycerol produced to sugar consumed ratio at the end of fermentations in JUICE, CRYO and JUICE + MARC.

	NT	LCC	HCC
**JUICE**	0.032 ± 0.002 *A*	0.033 ± 0.000 *A*	0.047 ± 0.005 *B*
	**NT**	**LCC**	**HCC**
**CRYO**	0.030 ± 0.000 *A*	0.041 ± 0.001 *B*	0.043 ± 0.000 *B*
	**NT**	**LCC**	**HCC**
**JUICE**+ **MARC**	0.030 ± 0.001 *A*	0.037 ± 0.002 *B*	0.041 ± 0.001 *B*

Data are expressed as the average of the replicates ± standard deviations. Different letters in each row indicate significant differences between values (*p* = 0.05). NT: untreated control, LCC: low cell concentration, HCC: high cell concentration.

## Data Availability

Data is contained within the article or supplementary material.

## Supplementary Materials

The following supporting information can be downloaded at: https://www.mdpi.com/article/10.3390/foods12010003/s1, Figure S1: Results of strain-specific primer PCR.
