# Supplementary material for: Starmerella bacillaris Released in Vineyards at Different Concentrations Influences Wine Glycerol Content Depending on the Vinification Protocols
Source: Foods. 2022 Dec 20;12(1):3. doi: 10.3390/foods12010003 (PMC9818441; doi:10.3390/foods12010003)
Supplement: Supplementary file 1 [file foods-12-00003-s001.zip › foods-1968563-supplementary.pdf]

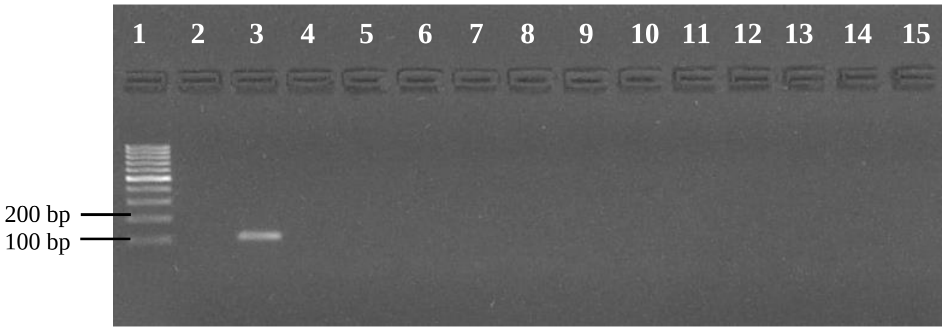

Figure S1. Results of strain-specific primers PCR. 1) molecular weight standard, Gene ruler 100 bp (ThermoFisher Scientific, Waltham, MA, USA), 2) negative control, 3) *Starmerella bacillaris* FRI751, 4) *Starmerella bacillaris* PAS13, 5) *Starmerella bacillaris* CBS9494, 6) *Starmerella stellata*, 7) *Candida oleophila*, 8) *Candida carpophila*, 9) *Candida intermedia*, 10) *Hanseniaspora uvarum*, 11) *Torulaspora delbrueckii*, 12) *Schizosaccharomyces pombe*, 13) *Saccharomyces cerevisiae*, 14) *Yarrowia lipolytica*, 15) *Metschnikowia pulcherrima.*
